# The association between PM2.5 exposure and suicidal ideation: a prefectural panel study

**DOI:** 10.1186/s12889-020-8409-2

**Published:** 2020-03-06

**Authors:** Yunsong Chen, Guangye He, Buwei Chen, Senhu Wang, Guodong Ju, Ting Ge

**Affiliations:** 1 Johns Hopkins University-Nanjing University Center for Chinese and American Studies, Gulou District, Nanjing, 210093 China; 2 School of Social and Behavioral Sciences, Nanjing University, 163 Xianlin Road, Qixia District, Nanjing, 210023 China; 3 The First Affiliated Hospital with Nanjing Medical University, Nanjing, 210029 China; 4 The University of Cambridge, 16 Mill Lane, Cambridge, CB2 1SB UK

**Keywords:** PM_2.5_, Suicidal ideation, Internet search

## Abstract

**Background:**

Suicidal ideation is subject to serious underestimation among existing public health studies. While numerous factors have been recognized in affecting suicidal thoughts and behaviors (STB), the associated environmental risks have been poorly understood. Foremost among the various environment risks were air pollution, in particular, the PM2.5. The present study attempted to examine the relationship between PM_2.5_ level and local weekly index of suicidal ideation (ISI).

**Methods:**

Using Internet search query volumes in Baidu (2017), the largest internet search engine in China, we constructed a prefectural panel data (278 prefectures, 52 weeks) and employed dynamic panel GMM system estimation to analyze the relationship between weekly concentration of PM2.5 (Mean = 87 μg·m^− 3^) and the index of suicidal ideation (Mean = 49.9).

**Results:**

The results indicate that in the spring and winter, a 10 μg·m^− 3^ increase in the prior week’s PM_2.5_ in a Chinese city is significantly associated with 0.020 increase in ISI in spring and a 0.007 increase in ISI in winter, after taking account other co-pollutants and meteorological conditions.

**Conclusion:**

We innovatively proposed the measure of suicidal ideation and provided suggestive evidence of a positive association between suicidal ideation and PM_2.5_ level.

## Background

Suicide has long been one of the most prominent and published public health problems, accounting for 800,000 deaths worldwide each year, according to the World Health Organization [1]. Far more common, however, are suicidal ideation and suicide attempts [1, 2]. In United States, each year, there are an estimated 25 million suicide attempts [3] and another 140 million individuals who have suicidal thoughts but haven’t acted on them yet, known as suicide ideators [4, 5]. In order to prevent suicide attempts and reduce suicide rates, the first and foremost is to identify potential population groups who are vulnerable to suicide. However, it is by no means easy, as Franklin et al. claimed in an extensive review of 365 suicide-related studies in the past 50 years [6]. Suicidal thoughts and attempts encompass a wide range of thoughts and behaviors, what’s most challenging is to identify the specific thoughts and behaviors closely associated with actual suicides. Note that, there is heavy stigma surrounding suicide, making the high-risk population reluctant to expose themselves. Internet search provide a way out, and such methods have already been widely used to reveal the potential attitude or behaviors that can hardly be captured using traditional methods [7–10].

While numerous factors have been recognized in affecting suicidal thoughts and behaviors (STB), such as, life stresses, chronic pain and illness, conflict, disaster, violence, losses, et al. [6], the environmental risks have been poorly understood. As a heatedly discussed dimension, air pollution has a detrimental impact on individual health, including higher rates of mortality [11–15], increased incidence of stroke [16], respiratory diseases [17–19], and lung and cardiovascular dysfunction [20–26], to name a few. In fact, air pollution has been revealed to be complicit in over 4 million deaths globally since 2015 [27]. Foremost among the pollutants associated with these mortality and morbidity numbers were airborne particulate matter including PM_10_ and PM_2.5_ (particles less than 10 or 2.5 μm in diameter). As a mixture of fine particles and liquid droplets, PM_2.5_ is a particular danger because it is easily sucked into the lungs, where it can have a devastating impact on an individual’s health.

Previous studies also indicate that PM_2.5_ and PM_10_ also have detrimental impact on mental health. For instance, depression has been reported by a bulk of literature to be closely correlated with PM_2.5_ [28–34]. Importantly, depression is a primary risk factor for suicide [35–38]. Therefore, it is likely that particulate matter such as PM_2.5_ and PM_10_ might also influence suicidal ideation. However, results of studies on the link between particulate matter and suicidal behaviors are mixed and mostly from western developed countries [39–42].

Since suicidal ideation is an independent and important risk factor for suicidal behavior [43], quantifying suicidal ideation in a scientific way and examining the ideation-related linkages should be a priority. There is a long chain of causality between particulates and suicidal behavior that begins with a series of shorter but clearer links between suicide-related mechanisms, such as particulate-depression, depression-ideation, particulate-ideation, depression-behavior, and ideation-behavior, etc. To determine if a relationship between ambient air particulates and suicidal ideation exists, we need to consider whether the variation of particulate matters in our research context (i.e., a given country, a city, or a society) is large enough to allow us to observe the differences that particulates make. This suggests that if findings obtained from industrialized societies are mixed, a more promising way of finding solid evidence is to look into developing countries where air pollution is more rampant.

Therefore, we chose China as the case for study. In the past two decades, China has experienced unprecedented economic growth accompanied by rapidly increasing mental health problems [44] as well as rapidly deteriorating air quality. The latest PM_2.5_ concentration data collected from the air quality monitoring station in more than 300 cities indicated that the majority of cities fail to meet air quality standards set by WHO [45]. Of note, the Chinese people and governments increasingly concerned with ambient air pollution due to the mass media and citizen watchdogs transmitting news and information about particulate matter (in particular PM_2.5_). Drastic actions have been taken to improve air quality in recent years including shutting down local coal-fired power plants, reducing the production of steel, and limiting driving. Still, the environmental hazard persists and makes China an ideal lab for looking into the potential link between suicidal ideation and ambient air particulates, especially PM_2.5_.

To quantify the aggregate level of suicidal ideation, we extract from Baidu (the largest online search engine in China) the local volumes of online search queries indicating suicidal thoughts manifested by the presence of plans to commit suicide. In fact, online searches indicating suicidal thoughts are used to quantify suicidal ideation in a recent study published in JAMA [46]. Besides, the closely related suicide-related internet search and suicide rates lend further support to the method of using online searches to measure suicidal ideation [47–50]. Compared with traditional survey methods and clinic processes to detect the extent of suicidal ideation, exploiting internet searching behavior has its unique advantages. Without the presence of a third party, suicide ideators are free to search for relevant information in private, which largely reduced the level of underreporting when using traditional survey method [51, 52]. The ideation-driven search behavior at the individual level aggregates and shapes the local pattern at the collective level. This suggests that in a city or county, the more suicide searches— “how to suicide,” “I want suicide,” “how to die easily,” or different ways to commit suicide—the higher the local suicidal ideation is.

Using this method to measure suicidal ideation, we proceed to examine whether and how local PM2.5 levels are associated with local suicidal ideation in China, based on nationally representative panel data collected from 278 Chinese prefectures in 52 weeks in 2017.

## Methods

### Research design

Using Internet search query volumes in China’s largest search engine Baidu and other online environmental data in 2017, we constructed a panel dataset at prefecture-level (278 cities, 52 weeks). In Baidu archives, most frequently searched items (words or short sentences) and the relevant IP locations from these searches were launched. Daily frequencies are computed as the Baidu Index. This allows us to access daily/weekly/monthly volumes of suicidal-ideation-related search queries at the prefecture level. We used Python 2.7.11 software to gather data on all chosen terms, and chose ‘week’ as the analytic time-frame unit over the course of 2017 because weekly time series are not affected by noise in daily time series and can be used to better identify seasonal and long-term trends of PM_2.5_ exposure and suicidal ideation.

### Index of suicidal ideation (ISI)

Prefecture-level suicidal ideation, the dependent variable, is gauged by the index of suicidal ideation (ISI) computed using suicide-related search data extracted from Baidu. As for the items signaling suicidal thoughts, we chose most commonly used phrases or short sentences clearly stating an intent to commit suicide. Specifically, we extracted the search volumes for five short sentences: 1) I want suicide (我想自杀); 2) I would like to commit suicide (我要自杀); 3) How to commit suicide (如何自杀); 4) suicidal methods (自杀方法); and 5) methods of suicide (自杀的方法); together with 11 Chinese words that can be seen as indirect signals of suicidal thoughts, such as various methods of committing suicide, the word suicide itself, and several synonyms. Specifically, these words are: 1) suicide (自杀), 2) wrist cutting (割腕), 3) hang oneself (上吊), 4) charcoal-burning (烧炭), 5) self-burning (自焚), 6) jump to death (跳楼), 7) cutting one’s own throat (自刎), 8) lay on the railway (卧轨), 9) attempt suicide (寻死), 10) suicide (*suicide*) and 11) Death ends all trouble (一了百了). The five direct and eleven indirect signal phrases/words together constitute the list of suicide-related words, based on which, we conduct principle component analysis (PCA). we choose the number of components based on the number of larger than one eigenvalue criteria. The PCA result shows that there is only one component whose eigenvalue is larger than one, in this regard, one principal component can capture most of variation of primary variables. By using regression method, we construct index of suicidal ideation ISI-1.

For a robustness check, we included only the five short phrases and dropped the eleven indirect signals of suicidal thoughts as these words may appear not only in individuals’ searches, but in news and fiction, which would overstate the degree of suicidal ideation. Note that there are obviously other phrases and words in Chinese representing suicidal thoughts, but the five we chose are the most searched and archived by Baidu. We perform the aforesaid PCA method to construct ISI-2. The results of PCA can be shown in Table 1.

**Table 1:** Factor loadings of depression search index in Baidu

Variables	ISI-1	Uniqueness	ISI-2	Uniqueness
“I want suicide”	0.681	0.537	0.742	0.449
“I would like to commit suicide”	0.415	0.828	0.498	0.752
“How to commit suicide”	0.695	0.518	0.741	0.451
“Suicidal methods”	0.67	0.551	0.731	0.465
“Methods of suicide”	0.661	0.564	0.728	0.471
suicide	0.768	0.41		
wrist cutting	0.712	0.492		
hang oneself	0.709	0.498		
charcoal-burning	0.556	0.691		
self-burning	0.453	0.795		
jump to death	0.733	0.463		
cutting one’s own throat	0.574	0.67		
lay on the railway	0.543	0.706		
attempt suicide	0.085	0.993		
suicide (suicide)	0.769	0.408		
Death ends all trouble	0.555	0.692		
Eigenvalue	6.186		2.412	
Variance (%)	38.66		48.23	
Cronbach’s Alpha	0.818		0.723	

*Note.* Principal component factor analysis. For ISI-1, KMO = 0.965 Bartlett’s test of sphericity = 5.23e+ 05 (120), *p* < 0.001. For ISI-2, KMO = 0.799 Bartlett’s test of sphericity =95,837.689 (10), *p* < 0.001

### PM_2.5_ and other co-pollutant measures

The key explanatory variable is a prefecture’s weekly average PM_2.5_ measured at 100 μg·m^− 3^ in 2017, which was collected from China’s Air Quality Online Monitoring and Analysis Platform (https://www.aqistudy.cn/historydata). We also collected the information of weekly average PM_10_, SO_2_, and NO_2_ from the same source, as various research has shown that these co-pollutants are associated with elevated risk of adverse health outcomes.

### Meteorological conditions

Additionally, the relationship between PM_2.5_ and suicidal ideation searches is likely to be confounded by time-variant factors. Previous studies that examined the effect PM_2.5_ on mental disorders and results such as suicide pointed out that confounding meteorological conditions including sunlight, temperature [53, 54], and rain fall should be taken into account [55–57] because these meteorological factors may simultaneously affect suicide risk [51, 58–63] and PM_2.5_ concentration. We controlled for weekly day mean temperature (*Temp*), weekly ratio of rainy days (*Rain*), and adjusted weekly sunlight (*Sunlight*) measured by the average length of a day in a given week multiplied by the weekly ratio of sunny days in each city over the course of the year. This information was collected from the Chinese website Historical Weather (http://www.tianqihoubao.com/lishi) and Sunrise and Sunset on the website of Convenience Inquiry (https://mtop.chinaz.com/site_richurimo.51240.com.html).

Given that the imbalanced internet development across regions may potentially influence individuals’ search behaviors, we further controlled for city-level frequency of surfing 11 of the most popular and widely-used web portals in China (*Webportal*). They are 百度 (Baidu), 微信 (We-chat), QQ (qq), 淘宝 (Taobao), 支付宝 (Alipay), 新浪 (Sina), 搜狐 (Sohu), 网易 (Netease), 腾讯 (Tencent), 中央电视台 (Chinese Central Television), and 高铁 (Bulliet Strain). To reduce the dimensionality of the data, we conducted principal component analysis and extracted one factor from these items. Moreover, we also controlled for the air pollution search to take into account the regional concern about air pollution. This measure not only reflected the regional population’s environmental awareness, but also reflected the media effect on air pollution. The descriptive statistics of key variables are shown in Table 2.

**Table 2:** Descriptive Statistics of Major Variables

Variable	Description	Mean	S.D.	Min	Max
ISI-1	Index of the suicide ideation measured by search volumes of indirect signals of suicidal thoughts from Baidu using PCA method (16 items)	0.016	0.967	−0.640	7.224
ISI-2	Index of the suicidal ideation measured by search volumes of five short sentences in Chinese from Baidu using PCA method (5 items)	0.014	0.891	−0.517	5.736
PM2.5	Prefecture’s weekly average *PM*_*2.5*_ 100 μg·m^− 3^ in 2017	0.862	0.499	0.189	8.670
PM10	Prefecture’s weekly average *PM*_*10*_ 100 μg·m^−3^ in 2017	5.416	3.120	0.330	44.460
SO2	Prefecture’s weekly average *SO*_*2*_100 μg·m^−3^ in 2017	1.224	1.159	0.020	19.480
NO2	Prefecture’s weekly average *NO*_*2*_100 μg·m^−3^ in 2017	2.092	1.018	0.120	6.860
WebPortal	Frequency of surfing Chinese major web portal	0.019	1.010	−0.933	11.408
PoSearch	Frequency of searching air pollution related items	0.019	0.889	−0.840	32.592
Temp	Weekly averaged day mean temperature	15.897	10.582	−23.900	34.786
Rain	Weekly ratio of rainy days (Rain)	0.265	0.243	0.000	1.000
Sunlight	Weekly sunlight	23.067	21.164	0.000	105.441

### Dynamic panel models

In this analysis, we take advantage of the panel to control for regional heterogeneity. To capture the dynamic structure of suicide-related searches, we adopted dynamic panel models by introducing the one-week lagged value (*t – 1*) of ISI to specify the path dependence condition. In order to deal with the reversed causality problem, we imposed the temporal order in the models by using the lagged values (*t – 1*) of all explanatory variables. The model can be written as:

1 $$ {ISI}_{i\_t}=\alpha {ISI}_{i\_\left(t-1\right)}+{\beta}_1 PM{2.5}_{i\_\left(t-1\right)}+{\beta}_2 PM{10}_{i\_\left(t-1\right)}+{\beta}_3 SO{2}_{i\_\left(t-1\right)}+{\beta}_4 NO{2}_{i\_\left(t-1\right)}+{\delta}_1{WebPortal}_{i\_\left(t-1\right)}+{\delta}_2{PoSearch}_{i\_\left(t-1\right)}+{\delta}_3{Temp}_{i\_\left(t-1\right)}+{\delta}_4{Rain}_{i\_\left(t-1\right)}+{\delta}_5{Daylength}_{i\_\left(t-1\right)}+{T}_i+{\mu}_i+{\varepsilon}_{it} $$

In the equation, *ISI*_*i* _ *t*_ is the dependent variable, representing the level of suicide searches in city *i* week *t*, while *ISI*_*i* _ (*t* − 1)_ is its one-week lagged value. *PM*2.5_*i* _ (*t* − 1)_ is the key independent variable, representing the level of weekly average *PM*_2.5_ in city *i* week *t* − 1. Considering the potential confounding from other co-pollutants and meteorological variables, we include one-week lag of *PM*_10_, *SO*_2_, *NO*_2_, temperature (*Temp*), rainy day ratio (*Rain*), weekly sunlight (*Daylength*) in city *i.* Moreover, to adjust for the different level of internet development and pollution concern across cities, one-week lag of frequency of searching air pollution related items (*WebPortal*) and frequency of searching air pollution related items (*PollutionSearch*) are also included in the Eq. (1). As shown, *β*_*i*_ and *δ*_*i*_ are the coefficients of the corresponding variables. *T*_*i*_ is the week-specific trend of suicidal ideation. *μ*_*i*_ is the city-level time constant error term, and *ε*_*it*_ is the city-level time-varying error term.

We employed System Generalized Method of Moments (SGMM) estimator to examine the association between PM_2.5_ and suicidal searches for three reasons. First, SGMM can rule out the impact of city-level heterogeneity in time-constant factors like fixed-effects models. Second, it can deal with the potential endogeneity problem by using appropriate lagged dependent and independent variable as “internal” instrumental variables. Third, by including the equations in both first-difference and levels [64, 65], we can downgrade the influence of measurement errors to a large degree [66]. We have conducted several diagnostic tests to check the validity of SGMM estimators. Specifically, we have performed: (1) the unit-root test and assure that all variables are stationary time series; (2) AR (2) test to check whether *μ*_*it*_ are serially correlated (*p* > 0.05); and (3) the Henson/Sargan over-identification test to examine the validity of these instruments (p > 0.05). Overall, the results of these tests suggest that SGMM is a valid estimator in this study.

## Results

### The trend of PM2.5 and ISI

**Fig. 1:**
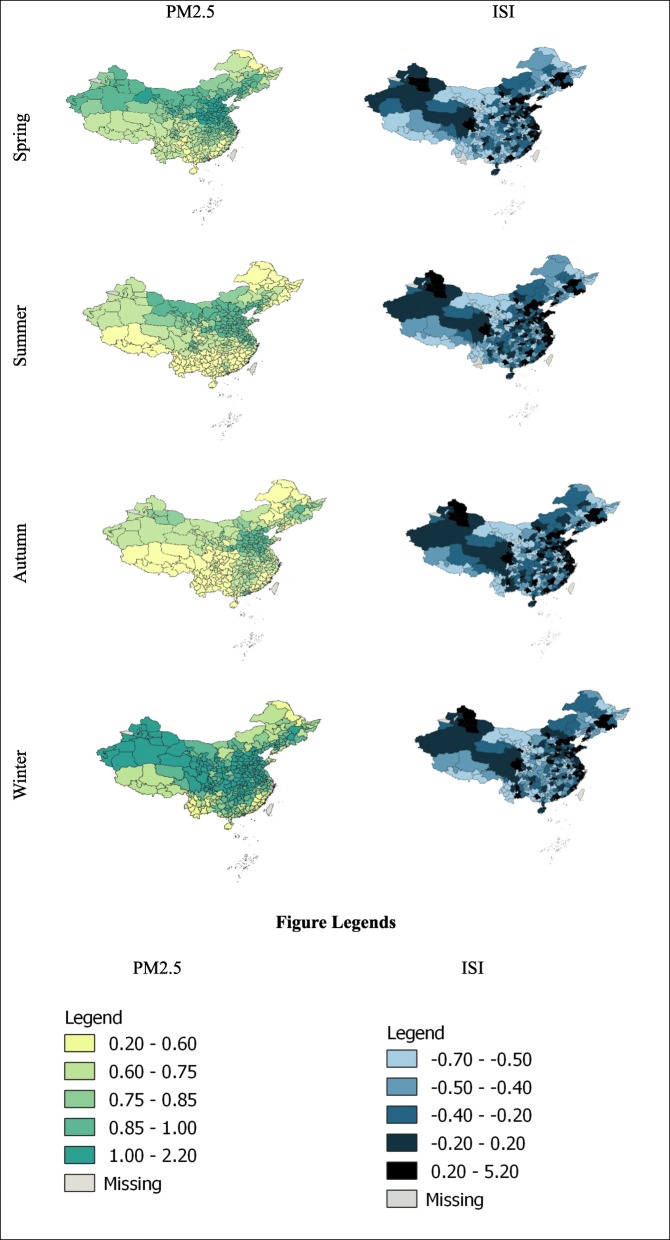
Weekly PM_2.5_ level and ISI in Chinese cities by season (2017). Note: The maps are generated using QGIS

Table 2 presents descriptive statistics for key variables in this study and shows the average PM_2.5_ level of all 278 cities over the course of the year was around 86.2 μg·m^− 3^. To show the distribution of PM_2.5_ and ISI across cities, we draw Fig. 1. As revealed, there was considerable regional variation in PM_2.5_ and ISI, and both varied seasonally as well. There is an elevated PM_2.5_ concentration in central China and some parts of northeast and northwest China where heavy industry abounds_._ However, in western, south and some parts of northeast and China where service industries prevail, PM_2.5_ concentration is relatively low. In terms of changes in PM_2.5_ concentration throughout the year, we observed a higher level of PM2.5 in winter than in other seasons. We also observed a larger variation in PM2.5 in northwest China relative to the rest of China, where a continental rather than a monsoon climate is dominant.

Table 2 also shows that average suicidal ideation of all 278 cities in 2017 was 0.016. The time series of provincial ISI is further drawn in Fig. 1. ISI is higher in most coastal areas, where internet use is more common. In some parts of Sichuan and northern Xinjiang, ISI is also high. This may be relevant to the high out-migration rate that leaves a large amount of left-over elderly, and children in that area. With regard to time trend, it is clear that, despite some seasonal changes, the variation of ISI is much smaller compared to PM_2.5_.

### Model results

In Table 3, we present model results of ISI-1, the measure of suicidal ideation by extracting the suicidal intention from five short phrases/sentences. The one-week lag of ISI is positive at the 0.001 significance level, meaning that ISI is the path dependent as expected. Web-Portal shows a significantly positive effect on ISI throughout the year. However, PM_2.5_ does not seem to significantly predict ISI. Given that the mixture of particulate matter varies by season, we further look into the effects across seasons.

**Table 3:** SGMM predicting suicidal ideation by season using ISI-1

	All	Spring	Summer	Autumn	Winter
Lagged ISI1	0.334^**^	0.198^***^	0.026	0.169	0.343^***^
	(0.102)	(0.031)	(0.104)	(0.243)	(0.040)
PM2.5	0.071	0.196^***^	−0.047	−0.044	0.069^*^
	(0.074)	(0.032)	(0.138)	(0.292)	(0.033)
PM10	0.002	0.014^***^	−0.002	−0.007	0.001
	(0.011)	(0.003)	(0.015)	(0.045)	(0.004)
SO2	−0.009	0.068^**^	−0.029	0.044	−0.010
	(0.103)	(0.022)	(0.111)	(0.105)	(0.019)
NO2	0.090	0.069^*^	−0.001	0.022	0.134^***^
	(0.069)	(0.027)	(0.089)	(0.203)	(0.021)
WebPortal	0.338	0.131	0.448^*^	0.457†	0.273^***^
	(0.293)	(0.107)	(0.190)	(0.236)	(0.062)
PoSearch	0.016	0.312^**^	0.174	0.144	−0.022†
	(0.049)	(0.098)	(0.222)	(0.175)	(0.012)
Temp	0.003	0.009^***^	0.014^*^	0.004	0.022^***^
	(0.004)	(0.002)	(0.006)	(0.003)	(0.003)
Rain	−0.000	0.000	−0.000	0.000	−0.002^+^
	(0.001)	(0.000)	(0.000)	(0.001)	(0.001)
Sunlight	−0.001	−0.001^*^	0.000	−0.000	0.000
	(0.001)	(0.000)	(0.001)	(0.003)	(0.001)
N	13,881	3539	3528	3544	1370
chi2	1766.110	450.296	301.061	310.205	447.354

*Note.* Numbers in parentheses are robust standard errors. *** *p* < 0.001, ** *p* < 0.01, * *p* < 0.05, † *p* < 0.1

The by-season results clearly show that, SO_2_ and NO_2_ do not exert significant impacts on ISI throughout the year. PM_2.5_ can positively predict ISI in spring and winter, even when taking account co-pollutants, and other weather conditions (e.g., temperature, rainfall, sunlight), regional internet usage, and pollution concerns. A 10 μg·m − 3 increase in PM_2.5_ is associated with a 0.020 increase of ISI in spring and a 0.007 increase in winter. Though PM_10_ also shows significant positive effect in spring, the effect is much smaller, merely 1/7 of PM_2.5_.

As a robustness check, we follow the same model-building strategy and take ISI-2, the measure of suicidal ideation, using more extended search items relevant to suicide as the dependent variable in Table 4. The results are consistent and only vary in the magnitude. Overall, this confirms that our results are robust regardless of measurement of ISI.

**Table 4:** SGMM predicting suicidal ideation by season using ISI-2

	All	Spring	Summer	Autumn	Winter
Lagged ISI1	0.488^***^	0.174^***^	0.032	0.433	0.384^***^
	(0.083)	(0.032)	(0.086)	(0.306)	(0.039)
PM2.5	0.076	0.286^***^	−0.024	−0.069	0.151^***^
	(0.076)	(0.038)	(0.049)	(0.263)	(0.038)
PM10	0.008	0.040^***^	−0.014	−0.001	−0.010
	(0.032)	(0.007)	(0.044)	(0.129)	(0.015)
SO2	−0.021	−0.045	− 0.148	0.074	− 0.025
	(0.142)	(0.074)	(0.423)	(0.210)	(0.053)
NO2	0.083	0.168^**^	0.104	−0.019	0.256^***^
	(0.174)	(0.064)	(0.349)	(0.347)	(0.037)
WebPortal	0.215^*^	−0.022	0.426^*^	0.306	0.048
	(0.098)	(0.156)	(0.177)	(0.287)	(0.070)
PoSearch	0.012	0.223^**^	0.258^*^	0.069	−0.017^*^
	(0.039)	(0.082)	(0.125)	(0.230)	(0.007)
Temp	0.003	0.010^***^	0.011^**^	0.005	0.010^*^
	(0.004)	(0.003)	(0.004)	(0.004)	(0.004)
Rain	0.000	0.000	−0.000	−0.000	−0.000
	(0.000)	(0.000)	(0.000)	(0.002)	(0.001)
Sunlight	−0.002†	−0.002^***^	− 0.000	−0.000	− 0.002^***^
	(0.001)	(0.000)	(0.001)	(0.002)	(0.001)
N	13,830	3526	3515	3531	3258
chi2	3228.240	279.557	469.719	550.914	747.642

*Note.* Numbers in parentheses are robust standard errors. *** *p* < 0.001, ** *p* < 0.01, * *p* < 0.05, † *p* < 0.1

## Discussion

Suicide is a global public health threat. Despite its importance, much remains unknown about the risk factors behind suicidal thoughts and behaviors. Some researchers blamed this to a lack of reliable STB indicators, leading to the slow progress and low effectiveness of suicide interventions [2, 43, 67]. In this study, to capture the potential suicide attempt, we take advantage of internet search data, a new method to measure the potential concern, or psychological tendency which cannot be captured in a traditional way.

In the past decades, numerous attempts have been made to establish risk factors of suicidal thoughts and behaviors, encompassing biological, psychological, and sociological factors [68–70], whether environmental factor is associated with suicidal thoughts and behaviors remain scare. Recent scholarship has shown that air pollution, specifically PM_2.5_, is a factor in depression. Suicidal ideation, a symptom of major depression is thus likely to be influenced by PM_2.5_. To explore such correlates, this research examines the potential link between PM_2.5_ and suicide ideation based on the context of China. The country has experienced rapid industrialization and modernization accompanied by an increase in mental health problems as well as environmental problems, where air pollution continues to pose a serious and sustained threat to residents physical and mental health.

We constructed a weekly index of suicidal ideation (ISI) in 278 major cities in China using internet searches that indicated suicide thoughts on Baidu, the most widely used search engine in China. The internet-based method of quantifying suicidal ideation allowed us to proxy the local level of thinking about suicide, which is often concealed, fleeting, and not easy to identify using traditional means. The SGMM estimators revealed that the effect of PM_2.5_ on suicidal ideation varies by season. A higher concentration of PM_2.5_ predicted a higher level of suicidal ideation in spring and winter, with ISI increasing for 0.020 in spring and 0.007 in winter for every 10 μg·m − 3 increase in PM2.5, holding constant of other co-pollutants and meteorological variables. For the rest of the year, PM_2.5_ did not show a significant effect. This result, to some degree, echoes the findings of depressive symptoms [71], where the peak prevalence of depression is usually discovered in winter [72–77] or spring [78, 79].

Overall, our study shows a significant and substantial positive relation between PM_2.5_ and suicidal ideation, contributing to the literature not only by shedding more light on our understanding of the pollution-suicide link but also by providing a scientific method to quantify suicidal ideation using online big data.

### Limitations and strengths

Our research has several limitations. First, due to stigmatization, individuals who are thinking of committing suicide incline to hide their true feelings, and often, they may express such inclination in a subtler and less straightforward way. In this vein, we may overlook some relevant searches that can signal suicidal thinking and behaviors. However, there are a wide range of thoughts and behaviors in suicidal thoughts and behaviors: thinking about suicide, developing a plan, obtaining the relevant tools. Only the last stage reflects a serious intention that may turn to physical action. In this vein, the search items we designed for this analysis captured intention to a large degree. Second, due to weekly changes in social, economic, and political conditions in each city, we may have ignored economic crisis, social conflict, or political hurdles that influenced suicide ideation. However, our analysis is based on weekly information in only 1 year, meaning that we focus on the short-term, rather than long-term impact of PM_2.5_. In this vein, assuming no short-term change of social, economic, and political conditions is plausible. Third, due to the lack of relevant clinical data, we cannot the identify the mechanism to establish the PM2.5-suicidal ideation link across seasons. However, as documented in various other research, the larger effect of PM_2.5_ in winter and spring is likely to be attributed to inflammation and oxidative stress [80–83], from high-emission of vehicle, biomass and coal combustion [84–88], for which, we will take it as a future direction to work on.

Despite these drawbacks, our research is an innovative attempt to quantify suicide ideation and reveal the potential linkage between PM_2.5_ and suicidal ideation. Our study shows a significant positive relationship between PM_2.5_ and ISI, contributing to the literature not only by shedding more light on our understanding of the pollution-suicide link but also by providing a new method to quantify suicide ideation using big data. The present environmental degradation and pollution is a product of rapid, unbridled industrialization. Amid public outcry over record-high pollution levels, researchers face a pressing urgency to understand how these pollutants are associated with physical and mental health risks.

## Conclusion

There is heavy stigma associated with suicide, making suicidal ideation a hidden pain. To reveal suicidal ideation as accurately as we can, we resort internet search data to construct a prefecture-level weekly index of suicidal ideation (ISI). By using this innovative measure, the present study examined the association between PM_2.5_ and ISI, and showed how such association would vary by season. Although future studies are still needed to explore specific mechanisms. Our findings might be taken as a reference for suicide prevention.

## Data Availability

All data are available from the authors.

## Abbreviation

ISI: Weekly index of suicidal ideation
